# Post-exposure testing at healthcare facilities with SARS-CoV-2 transmission: A rapid review

**DOI:** 10.4102/jphia.v16i2.623

**Published:** 2025-02-23

**Authors:** Emmanuel E. Effa, Okokon Ita, Joshua Mwankon, Funmi Siyanbade, Francis Iwomi, Eleanor Ochodo, Gemma Villanueva, Martin M. Meremikwu

**Affiliations:** 1 Department of Internal Medicine, Faculty of Clinical Sciences, University of Calabar, Calabar, Nigeria; 2 Department of Medical Microbiology, Faculty of Laboratory Medicine, University of Calabar, Calabar, Nigeria; 3 Department of Family Medicine, Faculty of Clinical Sciences, University of Calabar, Calabar, Nigeria; 4 School of Nursing, University of Calabar Teaching Hospital, Calabar, Nigeria; 5 Cochrane Nigeria, Calabar Institute of Tropical Disease Research and Prevention, University of Calabar Teaching Hospital, Calabar, Nigeria; 6 Centre for Global Health Research, Kenya Medical Research Institute (KEMRI), Kisumu, Kenya; 7 Cochrane Response, Cochrane, London, United Kingdom; 8 Department of Paediatrics, University of Calabar Teaching Hospital, Calabar, Nigeria

**Keywords:** SARS-CoV-2, post-exposure testing, healthcare facilities, transmission, systematic review

## Abstract

**Background:**

Post-exposure severe acute respiratory syndrome coronavirus 2 (SARS-CoV-2) testing following health facility outbreaks may control the spread of infection.

**Aim:**

This study aimed to assess the impact of testing for SARS-CoV-2 infection on health outcomes during healthcare facility outbreaks.

**Setting:**

This review included studies conducted at skilled nursing facilities, a cancer centre, and a geriatric psychiatric facility.

**Methods:**

We followed the methods for conducting rapid systematic reviews, searched databases from December 2019 to August 2022, assessed the risk of bias using the modified Newcastle Ottawa scale, and graded the certainty of evidence using the Grading of Recommendations, Assessment, Development and Evaluations (GRADE) approach. We pooled the prevalence, mortality, and hospitalisation results as appropriate.

**Results:**

Of the 3055 articles from database search, no study was eligible for inclusion as outlined in the protocol. However, eight non-comparative reports (case series) in skilled nursing facilities were included. The pooled prevalence of SARS-CoV-2 infection among residents of care homes and patients were 38% (95% confidence interval [CI] = 25% – 51%; 5 studies, 2044 participants; *I*^2^ = 94%, very low certainty evidence) and was 12% (95% CI = 6% – 19%; 5 studies, 2312 participants; *I*^2^ = 94%, very low certainty evidence) for exposed healthcare workers. The pooled mortality estimate and hospitalisation rate were 17% and 24%, respectively, (very low certainty evidence).

**Conclusion:**

There is no identified evidence for or against testing of people in healthcare facilities where there is ongoing transmission of SARS-CoV-2 infection.

**Contribution:**

The evaluation of the effectiveness of testing strategies during SARS-CoV-2 outbreaks need baseline and follow-up data from well-designed before and after studies appropriate for the setting.

## Background

Coronavirus disease 2019 (COVID-19), an infection with the severe acute respiratory syndrome coronavirus 2 (SARS-CoV-2) virus, has strained public health systems and healthcare facilities since its outbreak in December 2019. Globally, there have been approximately 600 million confirmed cases and 6.5 million deaths to date.^1^ Most of these deaths have occurred in the World Health Organization (WHO) regions of North America and Europe with disproportionately lower numbers in some low- and middle-income country regions. Among healthcare workers (HCWs), 152 888 infections and 1413 deaths were reported worldwide in the first year of the pandemic.^2^

Severe acute respiratory syndrome coronavirus 2 virus is spread within healthcare facilities through multiple routes ranging from respiratory droplets, personal items, private and public surfaces, as well as drainage systems.^3^ In addition, there is a higher risk of transmission on non-COVID wards when there is a delay in diagnosis in an exposed person and when HCWs lower their compliance with infection prevention and control (IPC) measures at nursing stations or tea rooms.^4^ Factors that may influence transmissibility following exposure, especially for at-risk individuals, include viral survival, dose-response rate, and the efficiency of spread from hands and surfaces to the respiratory tract. This is further influenced by the emergence of more transmissible variants of the SARS-CoV-2 virus variously described by the WHO as variants of interest (VOI) and variants of concern (VOC).^5^ The main VOC recently have been delta, omicron and omicron sub-variants. Of these, omicron and its sub-variants are reported to be more virulent, are more transmissible (even among the vaccinated), cause more severe disease, are more difficult to diagnose and treat and render public health and available social measures less effective.^5,6^ There are no currently circulating VOI, but previous ones such as epsilon, zeta, eta, theta, iota, kappa lambda and mu have posed increased community transmission risks and threats to global public health.^5^

Outbreaks of SARS-CoV-2 infections in healthcare settings have led to significant mortality among healthcare workers with a global population-based estimate of 115 500 deaths between January 2020 and May 2021,^7^ disruption of services and psychological effects on both patients and HCWs.^8^ Epidemiologic and genomic data suggest a higher likelihood of patient-to-patient transmission compared to staff-to-staff infections and that hospital-acquired infections result in more significant transmissions compared to community-acquired infections.^9^ Residents in long-term care facilities (LTCF) are a particularly vulnerable group to infection with, transmission of and mortality from SARS-CoV-2. In some regions of the world, LTCF residents experienced excess mortality from COVID-19 of between 30% and 40%.^10,11^ The risk of infection, transmission and severe outcomes is related to their older age, presence of chronic comorbidities, poor access to personal protective equipment (PPE), limited testing capacities, atypical symptoms and the recruitment of staff working at multiple facilities with the attendant risk of infection acquisition and spread within the facility.^12^

Public health containment strategies to limit the spread of the virus have included a combination of physical distancing, isolation of symptomatic persons, contact tracing, rapid mass population-based testing and vaccination.^13^ However, owing to the high transmissibility of the virus, especially recent variants in healthcare settings, IPC measures are necessary to prevent patients and HCWs from getting infected. These measures include universal masking for both patients and HCWs, hand hygiene practices, use of appropriate personal protective equipment by HCWs when giving care and screening before admission or procedures.^14^

Mitigation of the spread of SARS-CoV-2 within health facilities depends on the implementation of a combination of several WHO-recommended IPC strategies including: (1) standard, droplet and contact precautions; (2) cleaning and disinfection; (3) testing; and (4) engineering controls. Screening of patients and HCWs within a healthcare facility where there is a high risk of transmission of SARS-CoV-2 involves symptom-based screening and testing for the presence of the virus. However, the variability in the presymptomatic phases (persons who are symptom-free during a positive test but develop symptoms afterwards) and the high percentage of asymptomatic patients (persons with a positive test who never develop symptoms) make the use of symptom-based screening unreliable.^15^ Indeed, up to 30% – 45% of individuals infected with the virus have been reported to be asymptomatic.^16,17^

The detection of SARS-CoV-2 in infected persons is based on several molecular, immunoassay and imaging tests. Molecular tests detect viral ribonucleic acid (RNA) using manual or automated methods such as real-time reverse transcription polymerase chain reaction (rRT-PCR), which is one of several nucleic acid amplification tests (NAAT). Immunoassays are designed to detect viral antigens using immunodiagnostic techniques such as lateral flow assays (LFAs), also called rapid diagnostic tests (RDTs). Immunoassays also detect antibodies in those infected using serological techniques such as LFAs, enzyme-linked immunosorbent assays (ELISAs) or chemiluminescent immunoassays (CLIAs).^18,19^ Imaging tests include computerised tomography scan with specific SARS-CoV-2 abnormalities.^18^ However, the diagnostic accuracies of these tests are variable and depend on a number of factors including whether the exposed person is symptomatic or not.^18^

Prevention and control of outbreaks in healthcare settings rely on testing, which is a critical component of containment strategy for the COVID-19 pandemic. The availability of testing for exposed persons in healthcare settings should lead to quick identification of outbreaks, containment of spread and the proper institution of IPC measures such that vulnerable persons are protected from SARS-CoV-2 infection in the healthcare setting.

The WHO has developed guidance on testing of asymptomatic persons with a history of exposure to SARS-CoV-2-infected persons in various settings.^18,20,21^ In the course of the pandemic, recommendations for and against testing asymptomatic or exposed persons have been debated given the low diagnostic accuracies of available tests in asymptomatic and presymptomatic persons in various settings.^15^ The emergence of several more transmissible variants capable of causing more severe disease even among the vaccinated has led to the modification of rapid tests to improve their sensitivity.^22^ Despite this, the available point of care and RDTs have shown poor performance in detecting the virus in exposed and infected persons.^23^ This has necessitated the assessment of currently available evidence on the diagnostic effectiveness of testing exposed persons using available tests and the impact that may have on the outcomes of persons infected with the virus. Our objective was to assess the impact of testing for SARS-CoV-2 infection in people exposed to the infection in healthcare facilities on health outcomes.

## Methods

The protocol for this review was registered with the International Prospective Register of Systematic Reviews (PROSPERO) with the number CRD42022356551. We planned to include the following types of studies: randomised controlled trials, cluster and individual randomised controlled studies. Where RCTs were not found, we planned to include observational studies with a control group such as cohort studies (prospective or retrospective), case-control studies, controlled before-and-after study (CBA), historical control studies and interrupted time series (ITS). Ecological studies, clinical reports, outbreak reports and non-predictive modelling studies were to be excluded. However, in the absence of direct evidence, we have included case series that may provide indirect evidence.

### Types of participants

We considered studies that included people in facilities with suspected or confirmed SARS-CoV-2 transmission with a history of exposure to contact with SARS-CoV-2 as follows: residents in long-term care homes, in-patients (hospitals, etc.) including primary care levels, secondary care levels, tertiary care levels, outpatient care facilities, HCWs in long-term care homes, in-patient and out-patient facilities.

### Types of interventions

The interventions considered were testing upon exposure using any of the recommended diagnostic molecular or immunoassay (antigen or antibody) tests either as single tests or as a combination of tests such as rRT-PCR, LFAs, serological techniques such as LFAs, ELISAs or chemiluminescent immunoassays (CLIAs). The comparison was no testing upon exposure.

### Types of outcome measures

The primary outcome measures were SARS-CoV-2 infection in the facility, the proportion of tested participants with a positive test and all-cause mortality. The secondary outcomes were the proportion of positive participants treated for SARS-CoV-2, time from exposure to testing, participant satisfaction measured using a validated tool, and any adverse events. As this was a diagnostic effectiveness and not diagnostic test accuracy (DTA) review, we aimed to include DTA studies only if they reported qualitative data on the cost and user acceptability of post-exposure testing and planned to extract and present a narrative report of such information.

### Search methods for identification of studies

We searched the following databases from December 2019 to August 2022: The Cochrane Library – Central Register of Controlled Trials (CENTRAL) and Cochrane Database of Systematic Review, Medline (PubMed), EMBASE, the Effective Practice and Organisation of Care (EPOC), and LILACS. We also checked the reference lists of retrieved studies for additional reports of relevant studies. There was no language restriction. Full details of the search strategy are shown in Appendix 1. We used the Preferred Reporting Items for Systematic Reviews and Meta-Analyses (PRISMA) guideline and flow diagram to report the search and selection of studies.^24^

### Data collection and analysis

#### Selection of studies

The results of the search were distributed to four of the co-authors (F.I., O.I., F.S., J.M.) for screening. A more experienced author (E.E.E.) cross-checked a sample (30%) of the excluded studies for accuracy. Full reports of potentially relevant studies were obtained for further assessment. Two authors (F.I. and F.S.) independently applied the inclusion criteria to the full-text reports using an eligibility form and scrutinised publications to ensure each study was included in the review only once. We did not contact the study authors for additional information. We resolved disagreements through consensus agreement with the review team. We listed the excluded studies and the reasons for their exclusion in a table.

#### Data extraction and management

One author (E.E.E.) independently extracted data using a specifically developed piloted data extraction form. A second author (F.S.) cross-checked the extracted data for accuracy. We extracted where available data related to population, country, setting (residents of long-term homes, hospitals, outpatient, in-patient), age (adults, children), type of sample collected, type of test (rRT-PCR, antigen, antibody), frequency of testing/duration of intervention, outcomes reported, vaccination status, comorbidities, previous infections and variants for all included studies. We resolved disagreements through discussion between all review authors.

For each outcome, we extracted the number of participants exposed and the number tested in each facility.

#### Assessment of risk of bias in included studies

We had planned to assess the risk of bias of each included study using the appropriate ‘risk of bias’ tool for RCTs and resolve any disagreements by discussion between review authors. However, as we found no included studies as defined in our protocol, we decided to draw some indirect evidence from the case series. As the included studies were mainly case series, we used the modified Newcastle Ottawa scale and its modifications with eight items categorised into four distinct domains as follows: selection, ascertainment, causality and reporting.^25^ We categorised studies as low, medium or high risk of bias. The assessment is summarised in the risk of bias of the included studies table.

#### Measures of treatment effect

As heterogeneity was expected considering the included study design, variability in the participants and facilities and the lack of a comparator arm, we used an inverse-variance random-effects model to estimate the pooled prevalence, all-cause mortality as well as hospitalisation rates for both residents and HCWs and presented these results as forest plots indicating the effect estimates with their corresponding confidence intervals. We assessed statistical heterogeneity using the *I*^2^ statistic. We were unable to investigate clinical heterogeneity because of the small number of included studies. We were also unable to explore publication biases by constructing a funnel plot as there were not enough studies contributing to each outcome.

#### Strategy for data synthesis

We have combined studies in a meta-analysis for prevalence (residents, patients, and HCWs separately), all-cause mortality and hospitalisation rates using a random effect model and generated forest plots using the meta-prop syntax^26^ in STATA version 13.^27^

#### Assessment of certainty of evidence

We assessed the certainty of the evidence using the Grading of Recommendations, Assessment, Development and Evaluations (GRADE) approach.^28^ We appraised the certainty of the evidence for the important primary outcomes of SARS-CoV-2 infection (here reported as prevalence) and all-cause mortality and a secondary outcome, hospitalisation, derived from the included studies (each outcome against five criteria: risk of bias, consistency, directness, precision and publication bias).

### Ethical considerations

This article followed all ethical standards for research without direct contact with human or animal subjects.

## Results

**FIGURE 1:**
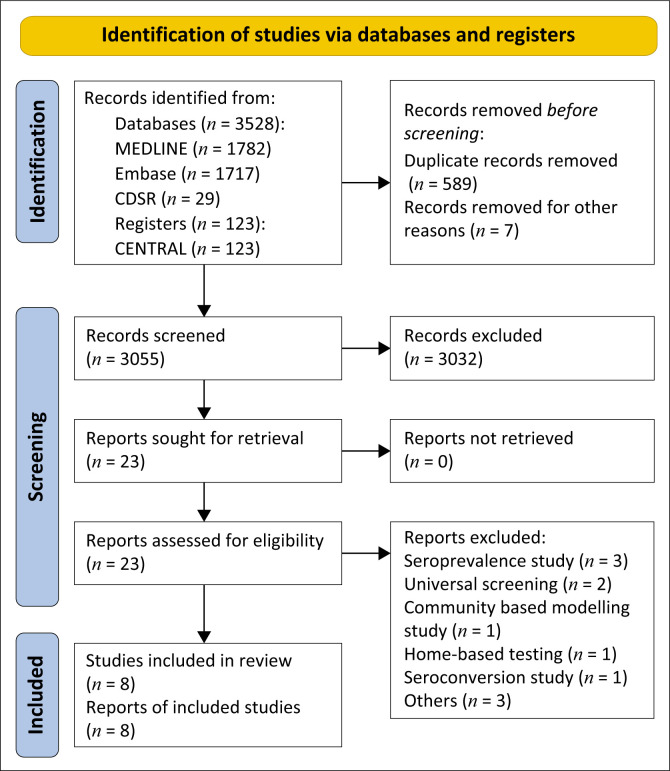
Preferred Reporting Items for Systematic Reviews and Meta-analyses (PRISMA) flow diagram.

The initial search returned 3528 articles and an additional three studies by searching the reference lists of included full-text articles. After removing duplicates, 3055 records were screened for eligibility. Of these, 3032 were excluded. There were no studies that met our inclusion criteria necessitating our consideration of case series to provide supportive information to draw indirect evidence from. Of the remaining 23 articles, 8 were included after reviewing their full texts (Appendix 1). This is shown in the PRISMA flow diagram (Figure 1).

### Characteristics of included studies

All eight included studies were case series. Six of the included studies were conducted in the United States.^29,30,31,32,33,34^ with a total of 2296 participants. One each was conducted in France^35^ and Japan^36^ and included a total of 136 and 111 773 participants, respectively. All the studies took place between March 2020 and June 2021 with the duration of the studies being between 1 month and 4 months. Full details are in the table of characteristics of included studies (Table 1).

**TABLE 1:** Characteristics of included studies.

Study ID	Country	Study period	Setting or facility	Total no. of participants	Methods	Participants	Interventions	Duration of intervention	Outcomes reported	Sample	Notes
Ariza-Heredia 2021^29^	US	March 2020 – April 2020	Cancer Centre	63	Case series	Healthcare workers	RT-PCR testing Test 2 days after outbreak	1 month	Proportion with COVID positive test	Nasopharyngeal sample	Symptomatic HCWs tested Data from cluster 1 only No new cases after 14 days
Arons 2020^30^	US	March 2020	Skilled Nursing Facility	89	Case series	Residents	RT-PCR testing and culture 6 days after the outbreak	1 month	Point prevalence, time to onset of symptoms, Doubling time of infections	Nasopharyngeal and oropharyngeal samples	Symptomatic and asymtomatic testing
Corcorran 2021^31^	US	March 2020 – May 2020	In-patient Geriatric Psychiatric Unit	81	Case series	Residents and healthcare workers	RT-PCR testing	2 months	Prevalence of SARS-CoV-2 infection Duration of SARS-CoV-2 RT-PCR positivity Death	Nasopharyngeal sample	25 patients 56 HCWs Reduction over the course of 2 weeks
Dora 2020^32^	US	March 2020 – April 2020	Skilled Nursing Facility for Veterans	232	Case series	Residents and healthcare workers	RT-PCR testing	1 month	Point prevalence, oxygen requirement, death, length of hospital stay	Nasopharyngeal sample	96 residents and 136 HCWs
Guery 2020^35^	France	April 2020	Medicalised Nursing Home	136	Case series	Healthcare workers	RT-PCR testing	1 month	Point prevalence	Nasopharyngeal	single testing
Patel 2020^33^	US	March 2020 – April 2020	Skilled Nursing Facility	126	Case series	Residents	RT-PCR testing	1 month	Point prevalence, hospitalisation, death	Nasopharyngeal	single testing
Shimizu 2022^36^	Japan	April 2020 – June 2021	Nursing Facility	111 773	Case series	Residents and HCWs	RT-PCR testing and loop-mediated isothermal amplification (LAMP) assays	3 months	Incidence rate ratios, hospitalisation, admission days, death	Not indicated	43 Long-term care facilities Screening tests were done within 1–2 days after the confirmation of the first case.
Telford 2020^34^	US	March 2020 – May 2020	Long-term Care Facilities	1705	Case series presented as response and preventive testing groups	Residents and Staff	RT-PCR testing	4 months	COVID-19 cases, hospitalisations and deaths	Nasopharyngeal	Included LTCFs in which facility-wide COVID-19 testing was initiated before identification of a COVID-19 case.

*Source:* Please see the full reference list of the article Effa EE, Ita O, Mwankon J, et al. Post-exposure testing at healthcare facilities with SARS-CoV-2 transmission: A rapid review. J Public HealthAfrica. 2025;16(2), a623. https://doi.org/10.4102/jphia.v16i2.623, for more information

COVID-19, coronavirus disease 2019; LTCF, long-term care facilities; HCW, healthcare worker; RT-PCR, reverse transcription polymerase chain reaction; SARS-CoV-2, severe acute respiratory syndrome coronavirus 2; US, United States.

### Setting

Most of the studies were conducted in long-term care and skilled nursing facilities. One study took place in a cancer centre,^29^ while another study was in a geriatric psychiatric centre.^31^

### Participants

In four of the studies, testing of both residents and HCWs commenced upon identification of a case within the facility,^31,32,34,36^ while in the other four studies, testing targeted either residents or HCWs alone.

### Intervention

Reverse transcriptase polymerase chain reaction testing was done for participants in all included studies using mainly nasopharyngeal samples. In addition, cultures were requested in one study,^30^ while loop-mediated isothermal amplification (LAMP) assays were done in another.^36^

In one study,^36^ the sample collected for testing was not indicated. Here, 43 long-term care facilities (LTCF) were included, and screening tests were done within 1–2 days after the confirmation of the first case. In another study^34^ involving participants in LTCFs, facility-wide COVID-19 testing was initiated before identification of a COVID-19 case in some of the facilities designated as ‘the preventive group’. We have only included data from ‘the response group’ where testing took place after an identified positive case. In only one included study was there data at baseline and 4 weeks after initiation of testing.^34^ In all studies, active case finding with testing was combined with transmission-based adjunctive IPC measures such as cohorting, isolation and heightened use of PPEs.

### Outcomes

All studies reported at least one of the primary outcomes. The number of participants positive for COVID-19 was reported in variable ways at the start of the screening. Point prevalence was the most often reported measure and was seen in five of the included studies.^30,31,32,33,35^ Two studies reported a proportion of COVID-19 positive,^30,34^ while one study reported incidence rate ratios.^36^ However, no study reported a proportion of COVID-19-positive participants at a specific time point after testing began. Mortality was reported in four studies.^32,33,34,36^ Other outcomes reported included time to onset of symptoms, doubling time of infections, hospitalisation and oxygen requirement.

### Risk of bias assessment and certainty of evidence

Five studies were classified as being of low risk of bias,^31,32,33,34,35^ two as medium risk of bias^29,30^ and one as high risk of bias.^36^ In the study with high risk of bias, the targeted participants for screening were determined by the capacity of the testing facility and the expected spread of COVID-19 within the facility based on an epidemiological survey.^36^ This may have excluded some potential participants. The details of the assessment are shown in the risk of bias table (Table 2). As all included studies were case series, the certainty of evidence for all assessed outcomes was rated as very low because of their inherent methodological limitations as shown in the summary of findings table (Table 3).

**TABLE 2:** Risk of bias.

S/N	Study ID	Selection	Ascertainment		Causality	Reporting	Overall risk
-	-	1. Does the patient(s) represent(s) the whole experience of the investigator (centre) or is the selection method unclear to the extent that other patients with similar presentation may not have been reported?	2. Was the exposure adequately ascertained?	3. Was the outcome adequately ascertained?	4. Was follow-up long enough for outcomes to occur	5. Is the case(s) described with sufficient details to allow other investigators to replicate the research or to allow practitioners make inferences related to their own practice	Low risk, Medium risk, High risk
1	Ariza-Heredia 2021^29^	1	0	1	1	1	Medium risk‡
	Support for judgement	Both symptomatic and asymptomatic HCWs were tested	There was no baseline data reported before the intervention was carried out.	Cases were identified by real-time reverse transcription polymerase chain reaction testing.	14 days after the last diagnosed case	A cluster of cases was defined as 2 or more cases of SARS-CoV-2– positive COVID-19 among HCWs who work in the same unit area at overlapping times. The identification of cases was carried out by testing symptomatic and asymptomatic HCWs with a reverse transcription polymerase chain reaction (RT-PCR) assay specific for SARS-CoV-2	
2	Arons 2020^30^	0	1	1	1	1	Medium risk‡
	Support for judgement	Residents in nursing home after an outbreak. Only residents that assented to be tested. Sample size not reported	Tests were done following a confirmed outbreak in the skilled nursing facility. It is unclear how many participants included in the study were exposed.	One-step real-time reverse transcriptase-polymerase chain reaction (rRT-PCR) on all samples and symptom-based checklist.	Follow-up over 3 weeks	The data was obtained from residents that assented Results of positive SARS-CoV-2 tests obtained during postmortem examination or by outside healthcare providers during clinical evaluation of symptomatic residents and staff were provided to the CDC and PHSKC through March 26.	
3	Corcorran 2021^31^	1	1	1	1	1	Low risk†
		All exposed patients and staff tested following SARS-CoV-2 infection in the facility	Two of the patients with SARS-CoV-2 symptoms were tested positive for the virus.	All samples were analysed using SARS-CoV-2 real-time RT-PCR assay or the Hologic SARS-CoV-2 real-time RT-PCR assay.	Repeat surveillance testing for SARS-CoV-2-positive patients was performed at 3- to 7-day intervals over 8 weeks.	All exposed (symptomatic/asymptomatic) persons tested and retested till negative.	
4	Dora 2020^32^	1	1	1	1	1	Low risk†
	Support for judgement	All facility patients and staff screened for SARS-CoV-2 following outbreak	Baseline exposure risk screening was done prior to the outbreak.	Nasopharyngeal samples were tested for SARS-CoV-2 by RT-PCR.	Testing was done over 4 weeks (March 29–April 23). Rounds of testing were done.	Reporting disaggregated for patients and health workers	
5	Guery 2020^35^	1	1	1	1	1	Low risk†
	Support for judgement	All 136 staff members, health workers, and administrative personnel (including auxiliary personnel) were tested.	Testing of staff members prompted following negative SARS-CoV-2 by RT-PCR but positive radiographic features in a patient	Nasopharyngeal RT-PCR tests	Testing was done over 2 days only.	Sufficient description of tested staff (all 136 staff members, health workers, and administrative personnel following a case)	
6	Patel 2020^33^	1	1	1	1	1	Low risk†
	Support for judgement	SARS-CoV-2 testing was offered to all residents of outbreak Facility A, regardless of symptoms. In addition, testing was offered to 70 staff members who worked on the ward where the index case lived.	Combination of exposure and symptom screening of residents and staff in outbreak facility who specifically worked on the ward with index case	Nasopharyngeal swabs for SARS-CoV-2 via RT-PCR	Residents were followed for 30 days (from 15 March to 14 April 2020) but no planned retesting.	Sufficient description of tested residents and staff including a flow diagram	
7	Shimizu 2022^36^	0	1	1	1	0	High risk§
	Support for judgement	The targets for screening were determined by the capacity of the testing facility and the expected spread of COVID-19 within the facility based on the epidemiological survey. This may have excluded some potential participants.	Screening tests were done on close contacts within one to 2 days after the confirmation of the first case in a nursing facility. Close contacts were not clearly defined.	PCR universal screening tests were performed at nursing facilities for the HCWs and users if a COVID-19 case was diagnosed within the facility.	Continuous tests were conducted over a period of 1 year. Re-infections were not indicated.	Definitions not consistently defined	
8	Telford 2020^34^	1	1	1	1	1	Low risk†
	Support for judgement	Testing of residents and staff members at 15 long-term care facilities conducted in response to a confirmed SARS-CoV-2 infection	Testing was carried out 1–5 days after identification of the index case. Residents and staff for testing identified through symptom-based screening.	Samples were tested for SARS-CoV-2 by real-time reverse transcription–polymerase chain reaction (RT-PCR).	A 1-day testing event for consenting residents and staff members was held at each LTCF from March 31–May 18. Symptom-based screening was conducted for 4 weeks thereafter to identify subsequent cases	Test groups are categorised as ‘response group’ and ‘preventive group’. Some were tested by private companies while some included presented evidence of positive results. Unclear what sample and what laboratory.	

*Source*: Murad MH, Sultan S, Haffar S, Bazerbachi F. Methodological quality and synthesis of case series and case reports. BMJ Evid-Based Med. 2018;23(2):60–63. https://doi.org/10.1136/bmjebm-2017-110853

COVID-19, coronavirus disease 2019; HCW, healthcare worker; RT-PCR, reverse transcription polymerase chain reaction; SARS-CoV-2, severe acute respiratory syndrome coronavirus 2; S/N, serial number.

† , Low risk;

‡ , Medium risk;

§ , High risk.

**TABLE 3:** Summary of findings – Severe acute respiratory syndrome coronavirus 2 testing compared to no testing for post-exposure to coronavirus disease 2019 disease.

Outcomes	Impact	No of participants (observational studies)	Certainty of the evidence (GRADE)
Prevalence of post-exposure SARS-CoV-2 infection among residents/in-patients (SARS-CoV-2 infection) assessed with: RT-PCR	The prevalence of SARS-CoV-2 infection among exposed residents of care homes/patients ranged from 19% to 64%. The pooled prevalence was 38% (95% CI = 25% – 51%; 2044 participants).	5	⨁◯◯◯ Very low†,‡, §
Prevalence of post-exposure SARS-CoV-2 infection among healthcare workers (SARS-CoV-2 infection among healthcare workers) assessed with: RT-PCR	The prevalence of SARS-CoV-2 infection among exposed healthcare workers ranged from 2% to 45%. The pooled prevalence of SARS-CoV-2 infection among exposed healthcare workers was 12% (95% CI = 6% – 19%; 2312 participants).	5	⨁◯◯◯ Very low¶, ††, ‡‡
Case-fatality rate (mortality)	The case-fatality rate ranged between 5% and 29%. The pooled case-fatality rate was 17% (95% CI = 5% – 30%)	4	⨁◯◯◯ Very low†,§,§§, ¶¶
Hospitalisation	Three studies reported hospitalisation of COVID-19 patients. In Patel 2020, 13 (37%) of 35 SARS-CoV-2-positive cases were hospitalised. In Aron 2020, 11(19%) of 57 SARS-CoV-2-positive cases were hospitalised. The pooled rate of hospitalisation was 24% (95% CI = 16% – 33%; 2 studies, 92 cases) among SARS-CoV-2-positive residents Dora (2020) reported a median length of hospital stay as 6 days (interquartile range 5 days to 10 days).	3	⨁◯◯◯ Very low†,§,¶¶

Note: Patient or population: Post-exposure to COVID-19 disease; Setting: Care homes and healthcare facilities; Intervention: SARS-CoV-2 testing; Comparison: no testing. GRADE Working Group grades of evidence: High certainty: we are very confident that the true effect lies close to that of the estimate of the effect. Moderate certainty: we are moderately confident in the effect estimate: the true effect is likely to be close to the estimate of the effect, but there is a possibility that it is substantially different. Low certainty: our confidence in the effect estimate is limited: the true effect may be substantially different from the estimate of the effect. Very low certainty: we have very little confidence in the effect estimate: the true effect is likely to be substantially different from the estimate of effect. The risk in the intervention group (and its 95% confidence interval) is based on the assumed risk in the comparison group and the *relative effect* of the intervention (and its 95% CI).

CI, confidence interval; SARS-CoV-2, severe acute respiratory syndrome coronavirus 2; RT-PCR, reverse transcription polymerase chain reaction.

† , At least one study (Aron 2020) was assessed to be at medium risk of bias;

‡ , Statistical heterogeneity was 94%;

§ , Included studies did not have a comparison ‘no testing’ arm;

¶ , At least one study (Ariza-Heredia 2021) was assessed to be at medium risk of bias;

†† , Statistical heterogeneity was 94%;

‡‡ , Included studies did not have a comparison ‘no testing’ arm;

§§ , Statistical heterogeneity was 72%;

¶¶ , Low sample size of cases.

## Primary outcomes

### Severe acute respiratory syndrome coronavirus 2 infection in the facility

**FIGURE 2:**
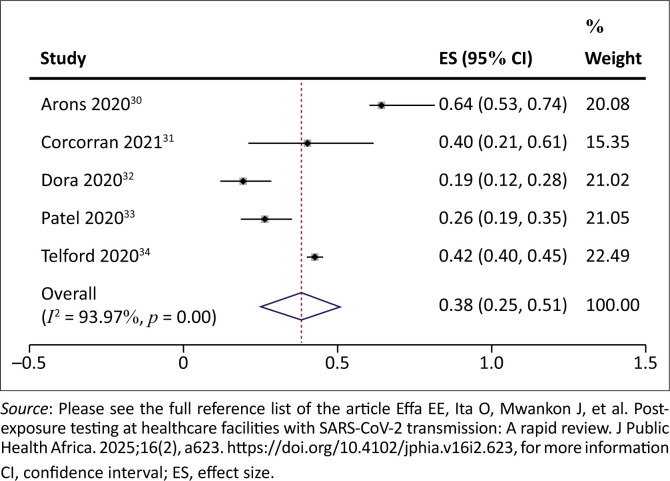
Prevalence of severe acute respiratory syndrome coronavirus 2 infection among exposed residents/patients.

No studies were identified that evaluated the impact of testing upon exposure to SARS-CoV-2 infection in the facility. However, we identified five studies reporting point prevalence of the infection in the facility. The prevalence of SARS-CoV-2 infection among exposed residents of care homes/patients ranged from 19% to 64%. The pooled prevalence was 38% (95% CI = 25% – 51%; 5 studies, 2044 participants; *I*^2^ = 94%, very low certainty evidence) (Figure 2). The results were downgraded to low certainty evidence because at least one study^30^ was assessed to be at medium risk of bias; there was significant statistical heterogeneity of 94%, and the studies were all observational as none had a comparison ‘no testing’ arm (Table 3).

**FIGURE 3:**
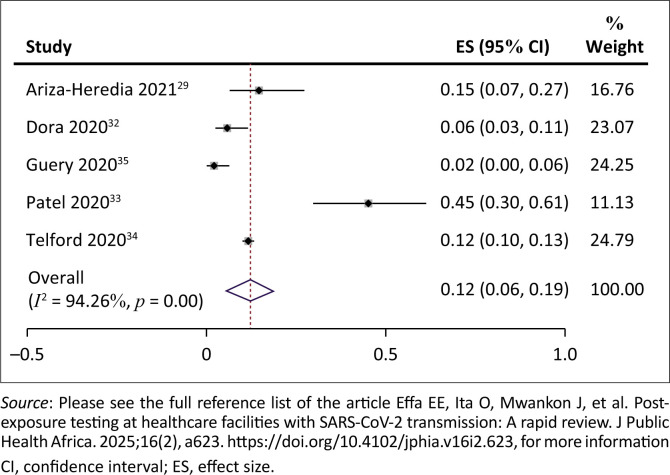
Prevalence of severe acute respiratory syndrome coronavirus 2 infection among exposed healthcare workers.

The prevalence of SARS-CoV-2 infection among exposed healthcare workers ranged from 2% to 45%. The pooled prevalence of SARS-CoV-2 infection among exposed HCWs was 12% (95% CI = 6% – 19%; 5 studies, 2312 participants; *I*^2^ = 94%, very low certainty evidence) (Figure 3). The evidence for this was rated as low certainty because at least one study^29^ was assessed to be at medium risk of bias; heterogeneity was significant (94%), and the included studies lacked a comparison ‘no testing’ arm.

In the only study with data at baseline and 4 weeks after initiation of testing, the prevalence was 28.0% and 42.4%, respectively, for residents and 7.4% and 11.8%, respectively, for healthcare workers.^34^

### All-cause mortality

**FIGURE 4:**
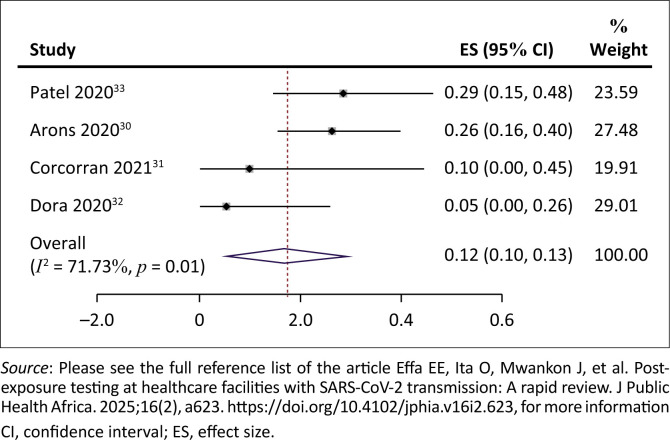
Mortality among severe acute respiratory syndrome coronavirus 2-infected residents/patients.

No studies were identified that evaluated the impact of testing upon exposure on all-cause mortality. However, we identified four studies that reported this on mortality rate.^30,31,32,33^ It was unclear if this was SARS-CoV-2-specific mortality or all-cause mortality. The case-fatality rate ranged from 5% to 29%. The pooled case-fatality rate was 17% (95% CI = 5% – 30%; 4 studies; 121 cases; *I*^2^ = 72%, very low certainty evidence) (Figure 4). The results were downgraded because at least one study^30^ was assessed to be at medium risk of bias, included studies did not have a comparison ‘no testing’ arm, statistical heterogeneity was 72% and sample size of cases was very low (Table 3).

## Secondary outcomes

None of the included studies explicitly reported on any of the secondary outcomes (proportion of positive participants treated for SARS-CoV-2, time from exposure to testing, participant satisfaction and adverse events) indicated in the protocol for this review.

**FIGURE 5:**
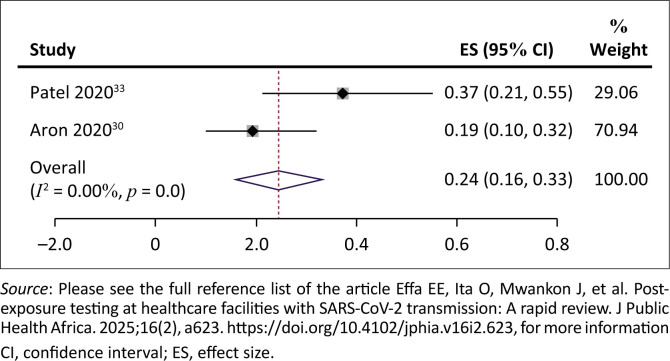
Rate of hospitalisation among severe acute respiratory syndrome coronavirus 2-positive residents.

However, three studies reported hospitalisation of SARS-CoV-2-positive residents of long-term care facilities. Hospitalisation was mostly described as admission to a formal hospital for additional care. In one study,^33^ 13 (37%) of 35 SARS-CoV-2-positive cases were hospitalised, while in another study,^30^ 11 (19%) of 57 SARS-CoV-2-positive cases were hospitalised. The pooled rate of hospitalisation was 24% (95% CI = 16% – 33%; *I*^2^ = 0%, very low certainty evidence) among SARS-CoV-2-positive residents (Figure 5). One study^32^ reported a median length of hospital stay as 6 days (interquartile range 5 days to 10 days). The results were downgraded because at least one study^30^ was assessed to be at medium risk of bias, included studies lacked a comparison ‘no testing’ arm and the sample size of cases was low.

## Discussion

In this rapid review on the effectiveness of deploying a testing strategy following an outbreak of SARS-CoV-2 infection in healthcare facilities to primarily mitigate spread, we found no eligible comparable studies to draw direct evidence from. We included case series in the review to provide indirect evidence to guide decisions. Although many of the included studies were of low risk of bias, they lacked any comparator arms leading to downgrading of the evidence to very low certainty across reported outcomes. Furthermore, all the studies were reported from high-income countries, so we cannot appropriately contextualise the findings. Lastly, most included studies have only provided baseline data which have been reported as prevalence but follow-up data to determine the effectiveness of the testing strategy is lacking.

The dynamics of transmission of SARS-CoV-2 in healthcare facilities has been affected by the emergence of new variants with differing transmissibility and the availability of vaccination.

Newer variants such as *omicron* and *delta* apart from being more transmissible are shed for longer periods after symptoms emerge.^37,38^ Indeed, emerging studies report no difference in virus shedding between vaccinated and unvaccinated individuals.^39^ These have implications for the timing of testing and periods of isolation of infected persons and may potentially disrupt service provision in places experiencing limitations in the number of health workers. The included studies were conducted at a time when vaccination was not available. Therefore, it is not possible to determine the relationship of the outcomes with vaccination status.

This review is limited by the inclusion of single-arm case series and the absence of results of testing from baseline and after the beginning of testing as well as the inclusion of data from only skilled nursing facilities where only elderly patients reside. The results cannot be generalised to regular hospitals with varying patient types.

## Conclusion

There is no identified direct nor indirect evidence for the effectiveness or not of testing people in healthcare facilities where there is ongoing transmission of SARS-CoV-2 infection.

## Appendix 1

**TABLE 1-A1:** Search strategy.

1.	exp Health facilities/ or exp hospitals/ or exp Nursing Homes/ or Homes for the Aged/ or assisted living facilities/
2.	exp Health Personnel/ or exp Health Occupations/ or Visitors to Patients/ or Adolescent, Hospitalized/ or Child, Hospitalized/ or Inpatients/ or Outpatients/
3.	((medical or health or healthcare) adj3 (facility or facilities or centre* or center* or office* or staff or personnel or occupation* or setting*)).ti,ab,kf.
4.	((health care or healthcare) adj worker*).ti,ab,kf.
5.	(hospital* or ward* or physician* or doctor* or surgeon* or surgical or surger* or dentist* or dental or nurs* or inpatient* or outpatient* or ambulatory or infirmar* or hospice* or Clinic or Clinics or “Old Age Homes” or “Old Age Home”).ti,ab,kf.
6.	((longterm care or long term care) adj3 (home* or facility or facilities or residence* or housing* or setting*)).ti,ab,kf.
7.	(exp Laboratories/ or Pharmacies/) not Health facilities/
8.	(Caregivers/ or Pharmacists/) not (Health Personnel/ or Health Occupations/)
9.	or/1-6
10.	or/7-8
11.	9 not 10
12.	exp covid-19 testing/ or covid-19 serological testing/ or covid-19 nucleic acid testing/
13.	((COVID* or SARS-CoV-2 or coronavirus* or COV or NCOV) adj7 testing*).ti,ab,kf.
14.	12 or 13
15.	exp Disease Transmission, Infectious/ or transmission/
16.	communicable disease control/ or infection control/
17.	(tm or pc).fs.
18.	(transmission or replication or prevent* or transmit* or spread* or contain or containment or proliferat*).ti,ab,kf.
19.	or/15–18 [*adapted from PubMed’s COVID-19 filters*]
20.	11 and 14 and 19
21.	randomized controlled trial.pt.
22.	controlled clinical trial.pt.
23.	randomized.ab.
24.	placebo.ab.
25.	randomly.ab.
26.	trial.ab.
27.	groups.ab.
28.	or/21-27
29.	exp animals/ not humans.sh.
30.	28 not 29 [*adapted from the Cochrane HSSS RCT filter*]
31.	20 and 30
32.	limit 20 to (meta-analysis or “systematic review”)
33.	exp cohort studies/ or exp epidemiologic studies/ or exp clinical trial/ or exp evaluation studies as topic/ or exp statistics as topic/
34.	((time and factors) or program or survey* or ci or cohort or comparative stud* or evaluation studies or follow-up*).mp.
35.	(control and (group* or study)).mp.
36.	or/33-35
37.	(animals/ not humans/) or comment/ or editorial/ or exp review/ or meta analysis/ or consensus/ or exp guideline/
38.	hi.fs. or case report.mp.
39.	or/37-38
40.	36 not 39 [*NRS filter by Waffenschmidt et al. 2020*]
41.	20 and 40
42.	31 or 32 or 41
43.	exp Animals/ not Humans/
44.	42 not 43
45.	(comment or editorial or newspaper article).pt.
46.	44 not 45
47.	exp health care facility/ or assisted living facility/ or exp hospital/ or nursing home/
48.	home for the aged/
49.	exp health care personnel/
50.	medical profession/
51.	patient visitor/
52.	exp hospital patient/
53.	outpatient/
54.	((medical or health or healthcare) adj3 (facility or facilities or centre* or center* or office* or staff or personnel or occupation* or setting*)).ti,ab,kw.
55.	((health care or healthcare) adj worker*).ti,ab,kw.
56.	(hospital* or physician* or doctor* or surgeon* or surgical or surger* or dentist* or dental or nurs* or inpatient* or outpatient* or ambulatory or infirmar* or hospice* or Clinic or Clinics or “Old Age Homes” or “Old Age Home”).ti,ab,kw.
57.	((longterm care or long term care) adj3 (home* or facility or facilities or residence* or housing* or setting*)).ti,ab,kw.
58.	or/47-57
59.	(exp laboratory/ or exp “pharmacy (shop)”/) not health care facility/
60.	(exp pharmacist/ or exp health educator/) not (paramedical personnel/ or health care personnel/ or health practitioner/ or paramedical profession/ or medical profession/)
61.	59 or 60
62.	58 not 61
63.	exp covid-19 testing/ or covid-19 nucleic acid testing/ or covid-19 serological testing/
64.	((COVID* or SARS-CoV-2 or coronavirus* or COV or NCOV) adj7 testing*).ti,ab,kw.
65.	63 or 64
66.	exp disease transmission/ or exp nosocomial transmission/ or exp pathogen transmission/
67.	communicable disease control/ or infection control/
68.	(transmission or transmit* or spread* or containment).ti,ab,kw.
69.	or/66-68
70.	62 and 65 and 69
71.	limit 70 to “systematic review”
72.	randomized controlled trial/ or controlled clinical trial/ or randomization/ or exp intermethod comparison/ or double-blind procedure/
73.	human experiment/
74.	(random$ or crossover or cross over or volunteer or volunteers or placebo or allocated or assigned or (open adj label)).ti,ab.
75.	(compare or compared or comparison).ti.
76.	((evaluated or evaluate or evaluating or assessed or assess) and (compare or compared or comparing or comparison)).ab.
77.	((double or single or doubly or singly) adj (blind or blinded or blindly)).ti,ab.
78.	parallel group$1.ti,ab.
79.	((assign$ or match or matched or allocation) adj5 (alternate or group$1 or intervention$1 or patient$1 or subject$1 or participant$1)).ti,ab.
80.	(controlled adj7 (study or design or trial)).ti,ab.
81.	trial.ti.
82.	or/72-81
83.	(Systematic review not (trial or study)).ti.
84.	(random cluster adj3 sampl$).ti,ab.
85.	(review.ab. and review.pt.) not trial.ti.
86.	(databases adj4 searched).ab.
87.	“we searched”.ab. and (review.ti. or review.pt.)
88.	“update review”.ab.
89.	Random field$.ti,ab.
90.	(nonrandom$ not random$).ti,ab.
91.	(random$ adj sampl$ adj7 (cross section$ or questionnaire$1 or survey$ or database$1)).ti,ab. not (comparative study/ or controlled study/ or randomi?ed controlled.ti,ab. or randomly assigned.ti,ab.)
92.	Cross-sectional study/ not (randomized controlled trial/ or controlled clinical trial/ or controlled study/ or randomi?ed controlled.ti,ab. or control group$1.ti,ab.)
93.	(((case adj control$) and random$) not randomi?ed controlled).ti,ab.
94.	(rat or rats or mouse or mice or swine or porcine or murine or sheep or lambs or pigs or piglets or rabbit or rabbits or cat or cats or dog or dogs or cattle or bovine or monkey or monkeys or trout or marmoset$1).ti. and animal experiment/
95.	Animal experiment/ not (human experiment/ or human/)
96.	(“cochrane database of systematic reviews” or “cochrane database of systematic reviews online”).jn.
97.	or/83-96
98.	82 not 97 [*Cochrane Embase RCT filter 2022 revision (Glanville et al. 2019)*]
99.	70 and 98
100.	Clinical article/ or controlled study/ or major clinical study/ or prospective study/ or cohort.mp. or compared.mp. or groups.mp. or multivariate.mp. [*NRS filter by Furlan et al. 2006 for Embase*]
101.	Controlled study/ or Treatment outcome/ or Major clinical study/ or Clinical trial/ or (chang$ or evaluat$ or reviewed or baseline or compare$ or compara$).tw. [*NRS filter by Fraser et al. 2006 for Embase*]
102.	case control study/
103.	100 or 101 or 102
104.	70 and 103
105.	71 or 99 or 104
106.	exp animal/ not exp human/
107.	105 not 106
108.	editorial.pt.
109.	107 not 108
110.	exp Health facilities/ or exp hospitals/ or exp Nursing Homes/ or Homes for the Aged/ or assisted living facilities/
111.	exp Health Personnel/ or exp Health Occupations/ or Visitors to Patients/ or Adolescent, Hospitalized/ or Child, Hospitalized/ or Inpatients/ or Outpatients/
112.	((medical or health or healthcare) adj3 (facility or facilities or centre* or center* or office* or staff or personnel or occupation* or setting*)).mp.
113.	((health care or healthcare) adj worker*).mp.
114.	(hospital* or ward* or physician* or doctor* or surgeon* or surgical or surger* or dentist* or dental or nurs* or inpatient* or outpatient* or ambulatory or infirmar* or hospice* or Clinic or Clinics or “Old Age Homes” or “Old Age Home”).mp.
115.	((longterm care or long term care) adj3 (home* or facility or facilities or residence* or housing* or setting*)).mp.
116.	(exp Laboratories/ or Pharmacies/) not Health facilities/
117.	(Caregivers/ or Pharmacists/) not (Health Personnel/ or Health Occupations/)
118.	or/110-115
119.	or/116-117
120.	118 not 119
121.	exp covid-19 testing/ or covid-19 serological testing/ or covid-19 nucleic acid testing/
122.	((COVID* or SARS-CoV-2 or coronavirus* or COV or NCOV) adj7 testing*).mp.
123.	121 or 122
124.	exp Disease Transmission, Infectious/ or transmission/
125.	communicable disease control/ or infection control/
126.	(transmission or replication or prevent* or transmit* or spread* or contain or containment or proliferat*).mp.
127.	or/124-126
128.	120 and 123 and 127
129.	46 use medal
130.	109 use emczd
131.	128 use cctr
132.	128 use coch
133.	129 or 130 or 131 or 132
134.	remove duplicates from 133
